# Supplementary material for: Concentrated Pre-Vulcanized Natural Rubber Latex Without Additives for Fabricating High Mechanical Performance Rubber Specimens via Direct Ink Write 3D Printing
Source: Polymers (Basel). 2025 Jan 28;17(3):351. doi: 10.3390/polym17030351 (PMC11820022; doi:10.3390/polym17030351)
Supplement: Supplementary file 1 [file polymers-17-00351-s001.zip › polymers-3419597-supplementary.pdf]

Supporting information

Concentrated pre-vulcanized natural rubber latex without additives for fabricating high mechanical performance rubber specimens via direct ink write 3D printing

Lin Liu[a, b], Jizhen Zhang[b], Zirong Luo[b], Na Kong[b], Xu Zhao[b], Xu Ji[a], Jihua Li[c], Shenbo Huang[b], Pengfei, Zhao[b], Shuang Li[b], Yanqiu Shao[a, *], Jinlong Tao[b, c*]

[a] Heilongjiang Key Laboratory of Photoelectric Functional Materials, College of Chemistry and Chemical Engineering, Mu Dan jiang Normal University, Mudanjiang, 157011, China

[b] Hainan Provincial Key Laboratory of Natural Rubber Processing, Agricultural Products Processing Research Institute, Chinese Academy of Tropical Agricultural Sciences, Zhanjiang 524001, China

[c] Rubber Research Institute, Chinese Academy of Tropical Agricultural Sciences, Haikou 571101, China

[*] Corresponding authors.

E-mail addresses: shaoyanqiu1969@163.com (Y.Q. Shao), jinlongt1983@163.com (J.L. Tao)

Table S1: The vulcanization system formula of pre-vulcanized natural rubber latex

Ingredients	Weight (kg)
Sulfur	0.86
ZnO	0.48
ZDC	0.77
PX	0.19
Antiaging agent-264	0.48
WLS	0.29
Casein	1.24
Diffusible agent-N	0.07
H_2O	1.79

Table S2: The formula of pre-vulcanized natural rubber latex

Ingredients	Weight (kg)
Casein	2
NR	160
H_2O	9
Substance of dispersion	6.2

In table 1, sulfur serves as an essential component for the preparation of pre-vulcanized latex. ZDC and PX function as accelerators, while Antiaging Agent-264 and WLS act as antioxidants.

Table S3: Comparative analysis of the mechanical properties of various 3D-printed elastomers.

Materials	Printing mode	Stress (%)	Strain (MPa)	Reference
SBR	VP	670	8.5	[1]
SiO_2-SBR	VP	432.07	8.8	[26]

SIR	VP	682	0.49	[27]
Styrene-butadiene rubber	VP	550	4.8	[28]
SiO_2-NPs	DLP	450	6.5	[29]
Liquid rubber	DIW	278.6	4	[30]
PUA-g-PEAs	LCD	256.7	5	[39]

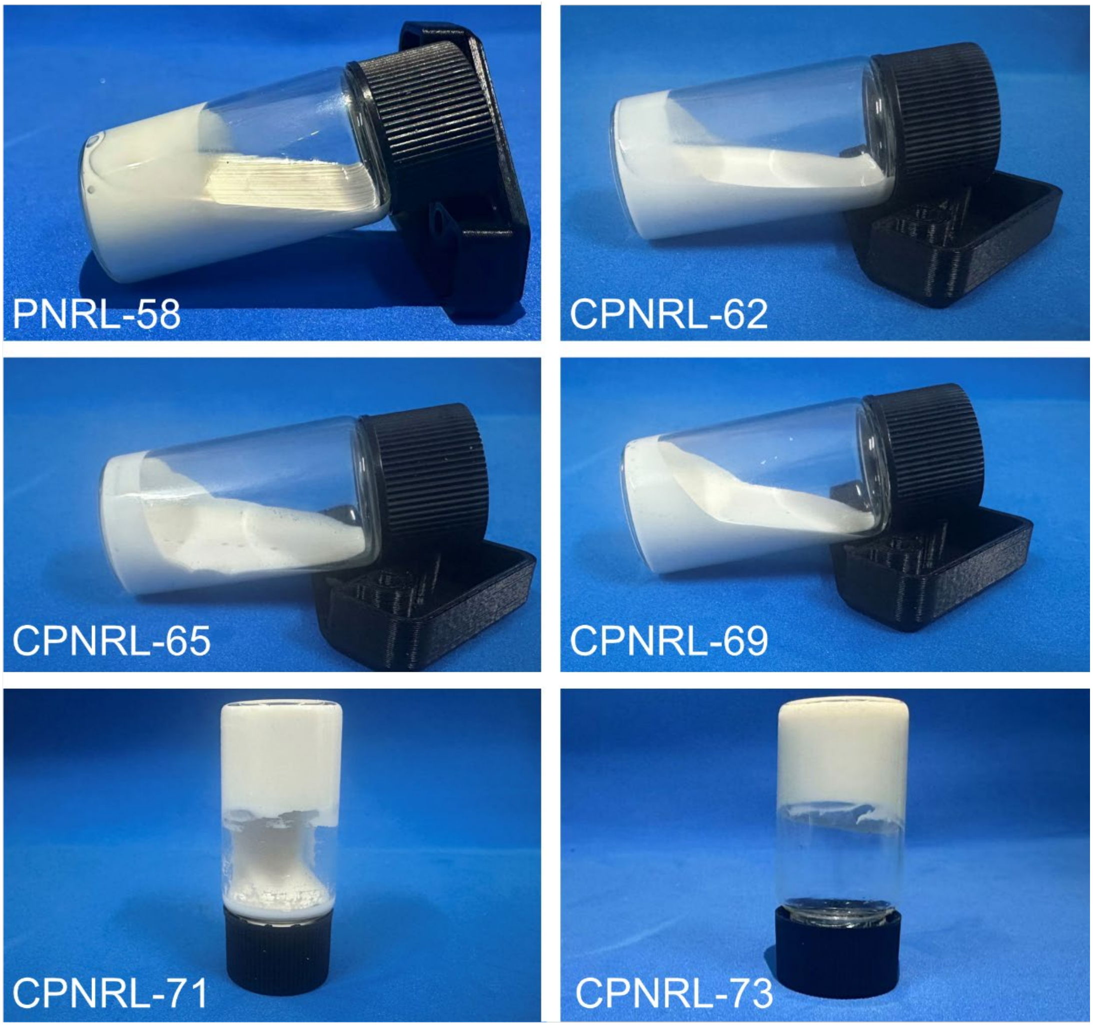

Fig. S1. Photographs of CPNRL with different solid contents.

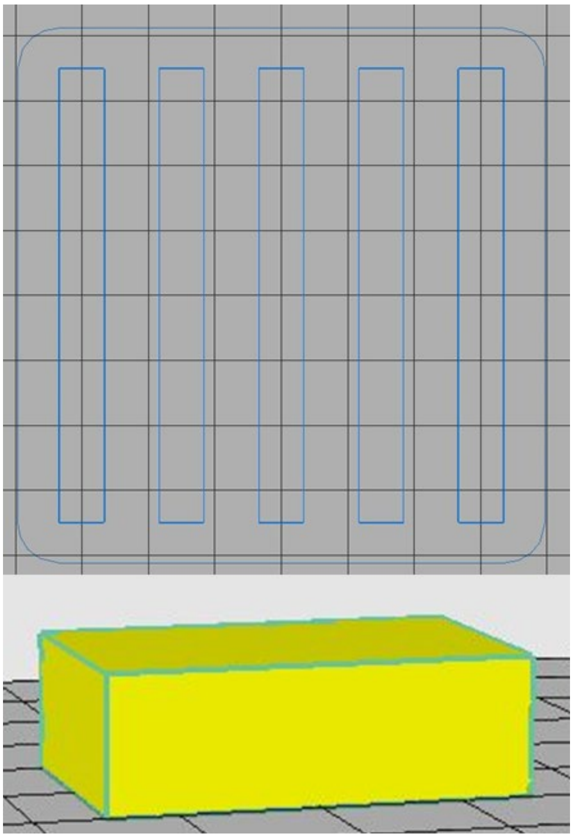

Fig. S2. Scale precision model of 3D-printed rubber sample
